# Barriers and facilitators to HPV vaccination of young women in high-income countries: a qualitative systematic review and evidence synthesis

**DOI:** 10.1186/1471-2458-14-700

**Published:** 2014-07-09

**Authors:** Harriet Batista Ferrer, Caroline Trotter, Matthew Hickman, Suzanne Audrey

**Affiliations:** 1 School of Social and Community Medicine, University of Bristol, Bristol BS8 2PS, UK; 2 Department of Veterinary Medicine, University of Cambridge, Cambridge CB3 0ES, UK

**Keywords:** Adolescents, Decision-making, HPV vaccine, Health inequalities, Ethnicity

## Abstract

**Background:**

Vaccination against Human Papillomavirus (HPV) is recommended for adolescent young women prior to sexual debut to reduce cervical cancer related mortality and morbidity. Understanding factors affecting decision-making of HPV vaccination of young women is important so that effective interventions can be developed which address barriers to uptake in population groups less likely to receive the HPV vaccine.

**Methods:**

We undertook a qualitative systematic review and evidence synthesis to examine decision-making relating to the HPV vaccination of young women in high-income countries. A comprehensive search of databases from inception to March 2012 was undertaken to identify eligible studies reporting the perspectives of key stakeholders including policy makers, professionals involved in programme, parents, and young women. Factors affecting uptake of the vaccine were examined at different levels of the socio-ecological model (policy, community, organisational, interpersonal and intrapersonal).

**Results:**

Forty-one studies were included. Whether young women receive the HPV vaccine is strongly governed by the decisions of policy makers, healthcare professionals, and parents. These decisions are shaped by: financial considerations; social norms and values relating to sexual activity, and; trust in vaccination programmes and healthcare providers. Financial constraints may be overcome through universal healthcare systems offering the HPV vaccine free at the point of delivery. In the healthcare setting, judgements by healthcare professionals about whether to recommend the vaccine may restrict a young woman’s access to the vaccine irrespective of her own beliefs and preferences. Parents may decide not to allow their daughters to be vaccinated, based on cultural or religious perceptions about sexual activity.

**Conclusions:**

Barriers to the uptake of the HPV vaccine have implications for young women’s future sexual, physical and reproductive health. Interventions to address barriers to uptake of the vaccine should target appropriate, and multiple, levels of the socio-ecological model. Issues of trust require clear, accessible, and sometimes culturally appropriate, information about the HPV vaccination programme. Although young women are central to the HPV vaccination programme, their views are underrepresented in the qualitative literature. Future research should consider young women’s perceptions of, and involvement in, consent and decision-making.

## Background

The World Health Organisation (WHO) recommends a three dose vaccination schedule for females aged between nine and 13 years [1] although schedules vary by country. Vaccination prior to sexual debut is advised to ensure protection before exposure to the target HPV types. In 2007, Australia became the first country to establish a national, school-based HPV vaccination programme offering HPV vaccine free at the point of delivery to young women aged between 12 and 13 years. Other countries, including the United Kingdom (UK), Sweden and Canada, have since introduced universal school-based HPV vaccination programmes within their national immunisation schedules. In the United States of America (USA), where insurance is the predominant model of care, the vaccine is provided through the healthcare setting; young women who are Medicaid eligible, uninsured or underinsured are eligible for vaccination free of charge [2].

The United Nations Convention on the Rights of the Child recognised the rights for all children and young people to participate in decision-making processes which involve them [3]. The legal framework for consent to vaccinate young women against HPV differs between countries. In Australia, parental consent is required and young women cannot be vaccinated without it. In contrast, in Canada, UK, USA and Sweden, young women are legally able to override parental decisions if they are considered mature enough to make, and understand the consequences of, the decision.

A systematic review and meta-analysis, comprising predominantly studies related to HPV vaccination programmes delivered in healthcare settings, indicated lower initiation by ethnic group and in young women without healthcare coverage insurance. No strong evidence for differences by parental education or family income variables were shown [4]. In the UK, where the HPV vaccination is provided primarily through the universal schooling system, inequalities in uptake by social deprivation have not been demonstrated [5-7]. However, lower uptake for minority ethnic young women are apparent [6-8]. This is of concern as women from minority ethnic population groups may be at increased risk of developing cervical cancer [9], but less likely to attend for cervical cancer screening [10,11].

High coverage of HPV vaccination programmes has the potential to reduce substantially cervical cancer incidence and mortality [12-14]. However, health inequalities may be increased if uptake remains lower amongst certain population groups. An increasing number of qualitative studies have been undertaken to provide insight into the views and perspectives from various population groups in relation to HPV vaccination of young women. By systematically retrieving, pooling and comparing the available data, qualitative synthesis can provide a better understanding of the reasons why some young women do not receive the HPV vaccine. This is important so that interventions can be developed to successfully promote uptake and address inequalities [15].

In order to provide understanding of factors affecting uptake of the HPV vaccination programme, we focused on facilitators and barriers to decision-making by key stakeholders. Additionally, explanations for lower uptake by young women from minority ethnic groups were sought.

## Methods

A protocol for this qualitative systematic review and evidence synthesis was not registered with a database. However, systematic review methods were followed to identify all the relevant qualitative literature pertaining to the research question and are described below.

### Search strategy

A comprehensive search strategy for Embase was developed to ensure that all relevant literature was captured. A combination of text words and the following indexing terms (MeSH) was used: “papillomavirus”, “wart virus”, “vaccination”, “immunization”, “immunization programs”, “wart virus vaccines”, “qualitative”, “interviews” and “focus groups” (Additional file 1). The search strategy was subsequently modified for other databases. The following databases were searched from inception to 9^th^ March 2012: CINAHL; Embase; Medline; PsycINFO, and; ISI Web of Science and ISI Proceedings. All abstracts were saved using Endnote ×3 reference manager software.

### Inclusion and exclusion criteria

Studies were eligible if qualitative research methods (interviews, focus groups, observations) or open-ended questions in questionnaires were used to explore views and behaviours related to decision-making of HPV vaccination of young women. The populations of interest were: young women; their parents and/or primary care givers; healthcare professionals involved in the delivery of the programme, and; other relevant stakeholders such as policy makers, community leaders, and teachers. Young women were defined as adolescent girls aged between nine and 18 years. Studies which included the views of women over the age of 18 years were included if the views of young women were reported separately.

No restriction was imposed by publication date in order to capture views of the HPV vaccine before its general availability (which might influence future delivery or uptake) as well as existing HPV vaccination programmes.

Studies not published in English were excluded. Conference abstracts, editorials, letters and books were included only if they presented original qualitative data. Primary studies with adults were excluded if views about HPV vaccination of young women were not reported in the results. Questionnaire studies reporting only closed questions were excluded. For this paper, with a focus on examining factors affecting decision-making in high-income countries, studies were categorised as high-income using the World Bank classifications [16].

### Study selection

Two reviewers (HF and SA) independently assessed the titles and abstracts from the literature searches and the relevance of studies retrieved as full text. Disagreements were resolved by discussion. The reference lists and bibliographies from relevant studies and reviews were hand-searched by one reviewer (HF) for additional primary studies not retrieved by the electronic search.

### Quality assessment

Currently, there is no consensus regarding the assessment of the quality of qualitative research and subsequent exclusion from systematic reviews [17]. For this systematic review and evidence synthesis, each primary study was appraised using the Critical Appraisal Skills Programme criteria for evaluating qualitative research [18]. Studies were not automatically excluded on the basis of overall ‘low quality’ if they contributed relevant qualitative information. However, the methodology and results of one study was presented in such a way that the findings were considered unreliable and this was excluded [19].

### Data extraction

Data pertaining to the methodology and context, including study and participant characteristics of each primary study, were extracted and entered into an excel spread sheet by one reviewer (HF).

### Thematic synthesis

Several methodologies for the synthesis of qualitative research exist [20].To analysis the data, the methodology was based on the methods of thematic synthesis reported by Thomas and Harden and using the Framework methods of qualitative data management [21-23]. These methods suit studies with *a priori* aims and objectives designed to directly inform policy and practice. The overall purpose of the synthesis was to ‘pool’ the results from individual primary studies by initially separating the findings, interpreting and then combining them through the identification of key themes across the studies [24].

Thematic synthesis was undertaken by one reviewer (HF) with discussions held with the second reviewer (SA) as analysis progressed. Elements of the text reported in the ‘results’ section of each primary study represented the basic units of the review. The text from each primary study was extracted verbatim and entered into a spreadsheet. The data was ‘charted’ into the matrix for studies relating to: young women, their parents/carers, and professionals.

Familiarisation with the dataset included reading and rereading the textual data in these primary charts. Sections of text were coded, with multiple codes being allocated where appropriate. The primary charts were retained and revisited as required, but streamlined versions were produced as the process of summarising and synthesising the data progressed. In these subsequent charts, key terms and phrases were retained while repetition within studies and extraneous text were removed. During this process, differences or similarities were identified within emerging themes.

### The socio-ecological model

During analysis the findings were considered in relation to the socio-ecological model [25] which considers that behaviour is shaped by a complex interaction between factors operating at the following levels: (i) public policy; (ii) community; (iii) organisational; (iv) interpersonal, and; (v) intrapersonal. We used the socio-ecological model to provide a framework for understanding how decisions of stakeholders at different levels of the model may affect access of the HPV vaccine for young women.

## Results

### Study characteristics

#### Search results

**Figure 1:**
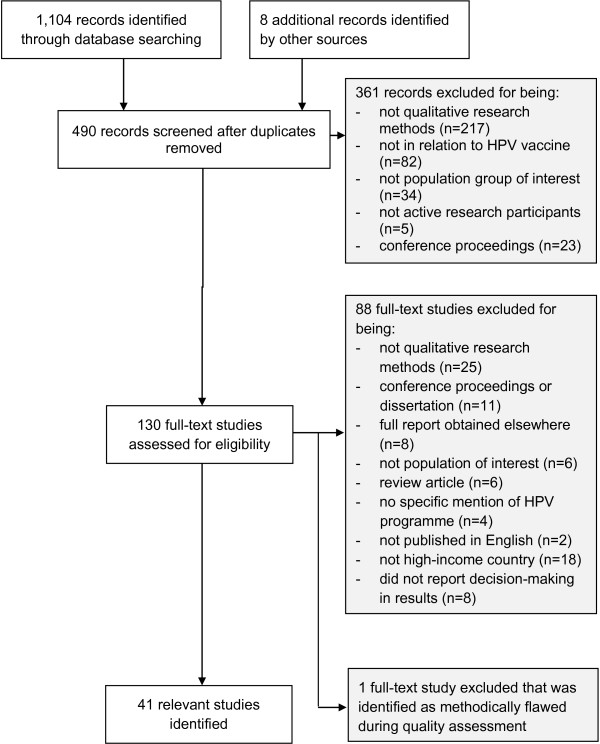
Flow diagram of study selection procedure.

Of 1,104 records initially identified through the database searches, 490 abstracts were reviewed and 130 full text articles assessed for eligibility of which 41 were eligible for inclusion (Figure 1).

#### Study and participant characteristics

Most studies were undertaken in the USA (n = 24, 58.5%) and the UK (9, 22.0%), with a further three studies in Australia, two in Sweden, two in Hong Kong and one in Canada. Study settings included healthcare (21, 51.2%), community (12, 29.3%), schools (6, 14.6%), government (1, 2.4%) and a combination of settings (1, 2.4%). Most studies were considered to be at moderate risk of bias (n = 20, 48.8%), followed by low risk (12, 29.3%), and high risk (9, 22.0%) (Table 1). Study participants included parents (16 studies, 39.0%), healthcare professionals (12, 29.3%), and young women (6, 14.6%), or a combination of participants (7, 17.1%). Reported sample size ranged from 10 to 522. More studies were undertaken post-licensure (25, 61.0%) of the HPV vaccine rather than pre-licensure (n = 16, 39.0%) (Table 2).

**Table 1:** Study characteristics of primary studies

**Study**	**Year**	**Country**	**Aim**	**Location**	**Type of setting**	**Data collection period**	**Data collection method**	**Sampling strategy**	**Analysis**	**Overall risk of bias**
Allen J, et al. [61]	2011	USA	To describe parents’ knowledge, attitudes, and decision-making with regard to obtaining the HPV vaccine for their daughters	Health and social service agencies, Boston	Community-based	February 2008 to May 2008	Focus groups	Convenience	Grounded theory	Medium
Bair R, et al. [51]	2008	USA	To describe Latina mothers’ acceptance of the human papillomavirus vaccine for their daughters and explore their knowledge base regarding HPV-related issues	One urban, paediatric primary care clinic	Healthcare-based	November 2004 to March 2005	Interviews	Convenience	Thematic analysis	Medium
Brabin L, et al. [53]	2007	UK	To investigate parents’ views on making available HPV vaccination to adolescent minors at sexual health clinics without parental consent	26 schools, city of Manchester	School-based	Not described	Semi-qualitative, questionnaire data	Convenience	Thematic analysis	Low
Brown E, et al. [55]	2009	UK	To explore GPs’ and practice nurses’ views of HPV vaccination, prior to implementation of the national immunisation programme, with a focus on their role and anticipated difficulties	Two general practices, Hampshire and Wiltshire	Healthcare-based	March 2008	Interviews	Convenience	Constant comparison and thematic analysis	Medium
Bynum S, et al. [31]	2009	USA	To explore adolescent girls and young women knowledge, beliefs and attitudes regarding HPV infection and vaccination, Pap tests, and cervical intraepithelial neoplasia	One teen clinic, Columbia, South Carolina	Healthcare-based	January 2007 to April 2007	Interviews	Purposive	Constant comparison	High
Chan Z, et al. [30]	2011	Hong Kong	To explore the experience and attitudes of physicians in clinics, and to facilitate physicians’ promotion of HPV vaccination	One district, Hong Kong	Healthcare-based	May 2010 to June 2010	Interviews	Convenience	Phenomenological approach	Medium
Colgrove J, et al. [23]	2010	USA	To identify the factors that were most influential in determining how states acted on the issue of mandates	Six states	Government-based	August 2008 to May 2009	Interviews	Purposive	Thematic content analysis	Medium
Constantine N, et al. [50]	2007	USA	To examine likelihood of parental acceptance of human papillomavirus vaccination for young adolescent girls, together with reasons for acceptance and non acceptance	Households, California	Community-based	March 2006 to September 2006	Semi-qualitative, questionnaire data	Random-digit-dial	Grounded theory	High
Cooper Robbins S, et al. [43]	2010	Australia	To explore experiences, knowledge, attitudes, decision-making processes, and contextual factors related to consent to vaccination and vaccination completion	Three schools, city of Sydney, New South Wales	School-based	September 2008 to June 2009	Focus groups, interviews and observations	Purposive	Constant comparison and thematic analysis	Low
Cooper Robbins S, et al. [64]	2010	Australia	To explore experiences, knowledge, attitudes, decision-making processes, and contextual factors related to consent to vaccination and vaccination completion	Three schools, city of Sydney, New South Wales	School-based	September 2008 to June 2009	Focus groups, interviews and observations	Purposive	Constant comparison and thematic analysis	Low
Dempsey M, et al. [49]	2009	USA	To compare the reasons why mothers do or do not have their adolescent daughters vaccinated against HPV	Outpatient family medicine or paediatrics clinics, University of Michigan’s healthcare system	Healthcare-based	January 2007 to March 2007	Interviews	Purposive	Content and thematic analysis	Medium
Do H, et al. [36]	2009	USA	To address HPV vaccine knowledge and beliefs among Cambodians living in Seattle, Washington	Seattle, Washington	Community-based	2008	Focus groups and interviews	Convenience	Thematic analysis	High
D’Souza C, et al. [37]	2011	Australia	To examine the development and delivery of a message targeting voluntary behaviour change	Northern Metropolitan local government region of Melbourne	Community-based	Not described	Focus groups	Not described	Health belief model	High
Friedman A, et al. [62]	2007	USA	To collect data on the general public’s knowledge, attitudes and beliefs regarding HPV and a hypothetical HPV vaccine and to explore their communication preferences	Six geographically dispersed sites.	Community-based	2003	Focus groups	Randomly selected	Thematic analysis	High
Gordon D, et al. [44]	2011	UK	To explore attitudes to human papillomavirus vaccination and reasons for accepting or declining the vaccine in the British Jewish community	Two Jewish schools, North London	School-based	June 2010 to September 2010	Interviews	Purposive	Framework analysis	Low
Gottvall M, et al. [39]	2011	Sweden	To investigate school nurses’ perceptions of HPV immunisation, and their task of administering the vaccine in a planned school-based program in Sweden	Five strategically chosen municipalities, Sweden	School-based	April 2010 to June 2010	Focus groups	Convenience	Content analysis	Medium
Hilton S, et al. [58]	2011	UK	To offer insights into adolescent girls’ understanding of HPV, its link with cervical cancer, and experiences of vaccination	Two regions in Scotland (Strathclyde and Lothian) and one region in England (London)	Community-based	December 2009 to May 2010	Focus groups	Convenience	Framework analysis	Low
Hilton S, et al. [42]	2011	UK	To offer insights from school nurses’ perspectives and experiences of delivering this new vaccination programme	National	Healthcare-based	September 2008 to May 2009	Interviews	Convenience and snowballing	Constant comparison	Low
Hughes C, et al. [41]	2011	USA	To generate hypotheses to inform interventions to increase vaccine receipt	Multi-state, hospital-owned, primary care practice based research network	Healthcare-based	March 2010 and June 2010	Interviews	Convenience	Grounded theory approach	Low
Humiston S, et al. [25]	2009	USA	To assess health care providers’ attitudes and practices regarding adolescent immunizations, including factors that either impede or facilitate vaccination	Monroe County, New York	Healthcare-based	2005	Focus groups and interviews	Not described	Grounded theory	Medium
Hutson S, et al. [60]	2011	USA	To investigate communication and cultural issues that may influence vaccine uptake among southern Appalachian women and explore their perceptions of HPV, cervical cancer and vaccination	Southern Appalachia	Community-based	October 2007 to August 2008	Interviews and focus groups	Convenience	Content analysis	Medium
Javanbakht M, et al. [28]	2012	USA	To explore healthcare providers perspectives on factors influencing HPV vaccination among adolescent girls in a community with high cervical cancer rates	Two clinics in Los Angeles	Healthcare-based	March 2009 to May 2009	Interviews	Convenience	Grounded theory approach	Medium
Kahn J, et al. [40]	2007	USA	To describe the range of pediatricians’ attitudes about human papillomavirus vaccines and to explore factors influencing their intention to recommend HPV vaccines	Three states of the United States (Ohio, Kentucky, and Indiana)	Healthcare-based	2005	Interviews	Purposive	Framework analysis	Low
Katz M, et al. [34]	2009	USA	To assess HPV vaccine acceptability	Ohio Appalachia counties	Community-based	Summer 2007	Focus groups and interviews	Convenience	Thematic analysis	Medium
Kwan C, et al. [32]	2008	Hong Kong	To explore perceptions towards cervical cancer, human papillomavirus infection and HPV vaccination and to identify factors affecting the acceptability of HPV vaccination among Chinese adolescent girls in Hong Kong	Community youth centre and secondary school	School and community-based	Not described	Focus groups	Purposive	Thematic analysis	Low
Marlow L, et al. [45]	2009	UK	To explore attitudes to HPV vaccination among black and Asian mothers living in Britain	Community health fairs and community groups	Community-based	April 2008 to August 2008	Interviews	Convenience and snowballing	Framework analysis	Medium
Mays R, et al. [47]	2004	USA	To elicit attitudes from parents on vaccinating children against sexually transmitted infections	Two paediatric clinics, Marion County, Indiana	Healthcare-based	January 2000 to June 2000	Interviews	Purposive	Content analysis	Medium
Mishra A, et al. [56]	2012	Canada	To illustrate the clinical, political and practical complexities of introducing an new and controversial vaccine	Not described	Healthcare-based	September 2009 to January 2010	Interviews	Convenience	Thematic analysis	High
Olshen E, et al. [46]	2005	USA	To explore parental views on the human papillomavirus vaccine	One urban, academic adolescent clinic and one suburban, private paediatric practice	Healthcare-based	September 2003 to March 2004	Focus groups and interviews	Convenience	Content analysis	Medium
Oscarsson M, et al. [38]	2011	Sweden	To explore youth clinic midwives role in cervical cancer prevention and their attitude to HPV vaccination	Two counties, Sweden	Healthcare-based	September 2010 to October 2010	Interviews	Convenience	Content analysis	Medium
Perkins R, et al. [29]	2010	USA	To explore parents’ opinions of school-entry requirements for human papillomavirus (HPV) vaccination	One urban, academic medical centre and an affiliated community health centre, Boston	Healthcare-based	June 2007 to February 2008	Interviews	Purposive	Content analysis and grounded theory	Medium
Perkins R, et al. [35]	2010	USA	To explore low-income minority parents’ attitudes, intentions, and actions with regard to human papillomavirus vaccination for their daughters	One urban, academic medical centre and an affiliated community health centre, Boston	Healthcare-based	June 2007 to February 2008	Interviews	Purposive	Content analysis and grounded theory	Low
Quinn T, et al. [27]	2012	USA	To conduct a qualitative examination of free text provider comments from a national survey of U.S. Physicians	National	Healthcare-based	2009	Semi-qualitative, questionnaire data	Not described	Grounded theory	Medium
Stretch R, et al. [57]	2009	UK	To seek the views of school nurses on vaccinating girls who did not have parental consent	Two primary care trusts, northern England	Healthcare-based	July 2008	Interviews	Convenience	Thematic analysis	Low
Shafer A, et al. [63]	2011	USA	To develop HPV vaccine messages for a campaign targeting racially diverse mothers of nonvaccinated 11 to 12 year olds	Rural Southeastern United States	Community-based	Not described	Focus groups	Not described	Constant comparison	High
Sussman A, et al. [59]	2007	USA	To better understand the implications for counselling about cervical cancer prevention by primary care clinicians who care for adolescents	New Mexico	Healthcare-based	July 2004 to May 2005	Focus groups	Purposive	Thematic analysis	Medium
Teitelman A, et al. [33]	2011	USA	To identify common beliefs about HPV vaccine initiation and describe the relationship between attitudes, norms, perceived control, and intention to receive HPV vaccine	Family planning, prenatal, or paediatric outpatient site for predominantly low-income patients.	Healthcare-based	Not described	Focus groups	Convenience	Content analysis	High
Tissot A, et al. [26]	2007	USA	To examine pediatricians’ views about key issues related to HPV vaccine delivery and identify their strategies for effective vaccine delivery	Three states of the United States (Ohio, Kentucky, and Indiana)	Healthcare-based	Summer 2005	Interviews	Purposive	Framework analysis	Low
Toffolon-Weiss M, et al. [48]	2008	USA	To describe Alaska Native parents’ knowledge of and attitudes towards cervical cancer, the human papillomavirus and the HPV vaccine	Three Alaskan communities	Community-based	January 2007 to March 2007	Focus groups	Convenience	Not described	High
Waller J, et al. [52]	2006	UK	To investigate responses to information about the HPV vaccine among mothers of daughters aged 8 to 14 years	Not described	Community-based	August and November 2005	Focus groups	Convenience and snowballing	Framework analysis	Medium
Williams K, et al. [54]	2010	UK	To explore knowledge about human papillomavirus and attitudes towards HPV vaccination among girls who were part of the ‘catch-up’ vaccination programme	One further education college and one state school	School-based	March 2009 to May 2009	Interviews	Purposive	Framework analysis	Medium

**Table 2:** Participant characteristics of primary studies

**Study**	**Year**	**Participants**	**Participant age (years)**	**Sample size**	**Vaccination Status of Young Women**	**Pre**-**licensure or Post**-**licensure period**	**Socioeconomic**	**Race/ethnicity**	**Sex**	**Religion**
Allen J, et al. [61]	2011	Parents of daughters aged 9 to 17 years old	Not described	64	Mixed	Post-licensure	Health insurance: 98%	Black: 59%, Hispanic: 19%, White: 23%	Female: 72%	Not described
Bair R, et al. [51]	2008	Latina mothers of daughters aged 7 to 14 years	Range: 24 to 40	40	N/A	Pre-licensure	Parental education: 38% reached 12th to 14th grade	Latina: 100%	Female: 100%	Not described
Brabin L, et al. [53]	2007	Parents of Year 7 (11 to 12 years old) students	46.6% aged 40 to 49	244	N/A	Pre-licensure	Free school meals: 26.4%	White: 67.6%; Black Caribbean: 9.4%; Black African: 7.4%; India sub-continent: 11.5%	Not described	None: 17.1%; Protestant: 47.1%; Catholic: 20.4%; Muslim: 7.5%
Brown E, et al. [55]	2009	General practitioners and practice nurses	Range: 28 to 56	17	N/A	Pre-licensure	General practices: one deprived area, two affluent area	Not described	Not described	Not described
Bynum S, et al. [31]	2009	Young women aged 14 to 20	Range: 14 to 20	68	Not vaccinated	Post-licensure	Public insurance: 64.6%	African American: 80.3%	Female: 100%	Not described
Chan Z, et al. [30]	2011	Physicians and general practitioners	Not described	12	N/A	Post-licensure	Not described	Not described	Not described	Not described
Colgrove J, et al. [23]	2010	Key stakeholders	Not described	73	N/A	Post-licensure	Not described	Not described	Not described	Not described
Constantine N, et al. [50]	2007	Parents with one or more daughter aged less than 18	70% of sample aged 30 to 49	522	N/A	Pre-licensure	Household income > $60,000: 43%	Non-Hispanic white: 41%, White: 38%, African-American: 7% Asian: 7%	Female: 73.4%	Catholic: 38.2%; Protestant: 14.0%; Other Christian: 17.7%; None: 13.6%.
Cooper Robbins S, et al. [43]	2010	Parents, teachers, vaccination nurses, and adolescents	Not described	185	Mixed	Post-licensure	Not described	Not described	All female, except one	Not described
Cooper Robbins S, et al. [64]	2010	Parents, teachers, vaccination nurses, and adolescents	Not described	185	Mixed	Post-licensure	Not described	Not described	All female, except one	Not described
Dempsey M, et al. [49]	2009	Mothers of vaccine eligible young women	Mean (vaccine declined): 41. Mean (vaccine received): 45	52	Mixed	Post-licensure	Some college/4-year degree: vaccine declined: 58%; vaccine received: 67%.	Not described	Female: 100%	Not described
Do H, et al. [36]	2009	Key informants and parents of at least one daughter eligible for HPV vaccine	70% of the sample aged > 40	50	Mixed	Post-licensure	Education < 12 years: 59%	American-Cambodian: 100%	Female: 51%	Not described
D’Souza C, et al. [37]	2011	School-age girls, youth centre attendees, and university participants	Not described	Not described	Vaccinated	Post-licensure	Not described	Australian: 80%	Female: 100%	Not described
Friedman A, et al. [62]	2007	Adults aged 25 to 45	Range: 25 to 45	314	N/A	Pre-licensure	Not described	Caucasian: 33%, Hispanic: 33%, African American: 33%	Female: 50%	Not described
Gordon D, et al. [44]	2011	Jewish mothers of vaccine eligible daughters	85% of sample aged 40 to 49	20	Mixed	Post-licensure	Degree education: 65%	Not described	Female: 100%	Jewish: 100%
Gottvall M, et al. [39]	2011	School nurses	Range: 35 to 60	30	N/A	Pre-licensure	Not described	Not described	Not described	Not described
Hilton S, et al. [58]	2011	Young women aged 12 to 18	Range: 12 to 18	87	Mixed	Post-licensure	High and low areas of deprivation	Not described	Female: 100%	Not described
Hilton S, et al. [42]	2011	School nurses	Not described	30	N/A	Post-licensure	Not described	Not described	Not described	Not described
Hughes C, et al. [41]	2011	Mother-daughter-physician triads	Young women range: 11 to 18	60	Mixed	Post-licensure	Mothers: education high school or less: 45%	Mothers: Black 60%, White 40%. Clinicians: Black 10%, White 75%.	Female: Clinicians 80%	Not described
Humiston S, et al. [25]	2009	Primary care practitioners	Not described	35	N/A	Pre-licensure	Not described	Not described	Not described	Not described
Hutson S, et al. [60]	2012	Women aged 18 to 50	Range: 18 to 49	39	Mixed	Post-licensure	Not described	Appalachian community	Female: 100%	Not described
Javanbakht M, et al. [28]	2012	Healthcare professionals	Not described	21	N/A	Post-licensure	Not described	Not described	Not described	Not described
Kahn J, et al. [40]	2007	Paediatricians	Range: 30 to 78	31	N/A	Pre-licensure	Not described	White: 58%, Black: 29%, Latino: 13%	Female: 55%	Not described
Katz M, et al. [34]	2009	Parents, community leaders, and healthcare providers	Range: 21 to 69	114	N/A	Pre-licensure	Various	White:106, Non-Hispanic: 111	Not described	Not described
Kwan C, et al. [32]	2008	Girls aged 13 to 20	Range: 13 to 20	64	N/A	Pre-licensure	Parental education: Secondary 64%	Chinese: 100%	Female: 100%	Not described
Marlow L, et al. [45]	2009	Black/Black British and Asian/Asian British mothers	Not described	20	N/A	Pre-licensure	Degree education: 50%	Asian: 50% Black: 50%	Female: 100%	Christian: 40%; Hindu: 10%; Muslim: 30%, no religion: 10%
Mays R, et al. [47]	2004	Parents with children aged 8 to 17	Range: 26 to 55	34	N/A	Pre-licensure	College: 50%	Not described	Female: 85%	Not described
Mishra A, et al. [56]	2012	Vaccine scientists and healthcare providers	Not described	15	N/A	Post-licensure	Not described	Not described	Not described	Not described
Olshen E, et al. [46]	2005	Parents	Mean: Urban participants; 40.5 Suburban participants; 44.7	25	N/A	Pre-licensure	Completed college: 32%	White: 44%, Black: 28%, Hispanic:16%	Female: 88%	Not described
Oscarsson M, et al. [38]	2011	Midwives	Range: 38 to 62	13	N/A	Post-licensure	Not described	Not described	Not described	Not described
Perkins R, et al. [29]	2010	Parents of vaccine eligible girls aged 11 to 18	Range: 31 to 60	73	Mixed	Post-licensure	Years of education: mean; 13	Caucasian: 26%, African-American: 25%, Afro-Caribbean or African: 21%, Latin; 29%	Females: 92%	Expressed religious affiliation: 82%
Perkins R, et al. [35]	2010	Low income parents	Range: 31 to 60	76	Mixed	Post-licensure	Years of education: mean; 13	Caucasian: 26%, African-American: 25%, Afro-Caribbean or African: 21%, Latin; 29%	Females: 92%	Expressed religious affiliation: 82%
Quinn T, et al. [27]	2012	Physicians	Not described	112	N/A	Post-licensure	Not described	Not described	Not described	Not described
Shafer A, et al. [63]	2011	Female caregivers of 11 to 12 year old girls	Not described	40	Not vaccinated	Post-licensure	Not described	African American: 58%, American Indian: 23%, Caucasian: 18%	Female: 100%	Not described
Stretch R, et al. [57]	2009	School nurses	Not described	15	N/A	Post-licensure	Not described	Not described	Not described	Not described
Sussman A, et al. [59]	2007	Paediatricians	Not described	37	N/A	Pre-licensure	Not described	Not described	Female: 86%	Not described
Teitelman A, et al. [33]	2011	Girls aged 13 to 26	Range: 13 to 26	34	Mixed	Post-licensure	Low income population	Black: 74%	Female: 100%	Not described
Tissot A, et al. [26]	2007	Paediatricians	Mean: 46.9	31	N/A	Pre-licensure	Not described	White: 58%, Black: 29%, Asian American: 7%	Female: 55%	Not described
Toffolon-Weiss M, et al. [48]	2008	Native Alaskan parents of adolescents aged 9 to 18	38% aged between 41 and 50	80	Mixed	Post-licensure	Not described	Alaskan natives	Female: 81%	Not described
Waller J, et al. [52]	2006	Mothers of girls aged 8 to 14	Range: 31 to 48	24	N/A	Pre-licensure	Degree level: 50%,	Not described	Female: 100%	Not described
Williams K, et al. [54]	2010	Girls aged 17 to 18	Range: 17 to 18	10	Mixed	Post-licensure	Not described	White British: 80%; British Asian: 20%	Female: 100%	None: 80%; Muslim: 20%

#### The socio-ecological model and cross cutting themes

The data suggest that young women’s access to the HPV vaccine is influenced by the overall policy context and decisions of key stakeholders operating at different levels of the socio-ecological model including healthcare professionals and teachers, parents and the young women themselves (Table 3). Five cross cutting themes were also identified that related to decision-making: mandate, finance, sexual mores, trust, and consent. These are discussed below from the perspectives of the different stakeholders involved. Although these themes are relevant to all young women, it became clear that some had particular pertinence for young women from minority ethnic groups and may help to explain identified differences in uptake. This is considered further in the discussion.

**Table 3:** The socio-ecological model: factors influencing young women’s uptake of the HPV vaccine in high-income countries

**Level**	**Key issues**	**Cross cutting themes**				
Policy	Vaccine availability, cost and delivery	Mandate	Finance	Sexual mores	Trust	Consent
Community	Social norms and values					
Organisational	Healthcare professional recommendation and provision					
Interpersonal	Parental decision-making and consent					
Intrapersonal	Young women’s characteristics and consent					

#### Mandate

In the USA, routine HPV vaccination was recommended for girls between ages 11 and 12 by the national Advisory Committee on Immunisation Practices (ACIP) in 2006 [26]. However, whether a vaccine should be mandatory for school attendance is predominantly decided by state legislatures and is subject to debate. At the time the studies were undertaken, policy makers in the USA were not clear that a school-based mandate for the HPV vaccine was appropriate. Lack of transmissibility in the school-setting was felt to undermine the need to a mandate: “*I can completely support it in certain kinds of infectious diseases that are a threat in terms of morbidity and mortality*, *and are easily transmitted within a classroom*, *for example*, *but HPV is not one of the things*” [Policy maker, USA] [27]. Policy makers also considered likely opposition from ‘anti-vaccination’ groups [27,28].

The mode of transmission of HPV was also considered by healthcare professionals [29,30]: “*The only mandates we currently have for vaccines in our country are for infectious diseases spread in a school setting. It would not be a school issue or a public health issue*, *but more of an individual issue*” [Paediatrician, USA] [30]. However, school-entry mandates were also perceived by healthcare professionals to be advantageous in creating universal access to HPV vaccine: “*Realistically*, *that*’*s how you*’*re going to get those patients who fall under the system to get vaccinated*” [Paediatrician, USA] [30]. Further, the absence of school-entry mandates was thought to diminish parental perceptions of the importance of the vaccine [29,31,32]: “*Some patients*, *again*, *come in saying*, ‘*We don*’*t want that because*’ *friends of them are telling them it*’*s not really mandatory or it*’*s not really gonna affect* ‘*em*” [Staff person, USA] [32].

Some parents shared the concerns of policy makers and healthcare professionals, in relation mandating a vaccine for a disease that was not transmissible in the school setting [33]. However, other parents supported a school-entry mandate to ensure that all young women could receive benefits of the vaccine: “[*If HPV vaccine were mandated*, *I would feel*] *grateful because we parents want the best for our children*, *and thank God science has discovered new medicines to prevent diseases*” [Latina mother, USA] [33].

#### Finance

In countries without universal healthcare coverage, the costs of the vaccine were identified as an important barrier to provision of, and access to, the HPV vaccine. In the USA, policy makers considered that public bodies and private insurance companies had been responsive in meeting HPV vaccine associated costs [27]. However, healthcare providers noted a financial burden that influenced whether they provided vaccines to their population: “*There is no way vaccines are cost*-*effective for us. It costs us an incredible amount of money in terms of time. Because the time we spend*, *the time the nurses spend*…*we*’*re not even close to being reimbursed for the amount of time we spend on vaccines*” [Practitioner, USA] [29].

Without universal healthcare coverage, the financial costs of the HPV vaccine appear likely to prevent uptake by the typically most disadvantaged families. Healthcare professionals indicated the high price [34], lack of healthcare insurance [30,32], and inadequate insurance reimbursement [29,31] were important barriers to provision and uptake: “*The only reason myself and my colleagues* [*sic*] *not able to offer HPV vaccine to our patients is lack of reimbursement* [*by insurance companies*]” [Physician, USA] [31].

Financial barriers were also mentioned by families and community members in these settings [35-40]: “*My gynecologist offers the HPV vaccine but you have to pay* $*135 up front and it is three shots*, *so you pay* $*135 a piece for each shot and insurance does not reimburse you or cover it*” [Participant, USA] [38];“*Insurance don*’*t pay for everything* [*provides partial payment*] *and then a lot of times insurance doesn*’*t cover everything* [*some services not covered at all*]” [Young woman USA] [37]. Where the vaccine was not available free at the point of delivery, young women were also aware that the high cost could render the HPV vaccine unaffordable [35-37]:“*Where will I get the money for it* [*HPV vaccine*]? *At this price*, *my family could not support me either*” [Young woman, Hong Kong] [36]. This contrasts with the ‘catch up’ campaign in Australia where the vaccine was provided free for a limited time: “*They were doing it for free*, *so mum was like*, ‘*You*’*d better go*’ ” [Young woman, Australia] [41].

In Sweden, the HPV vaccine was made available in the healthcare setting for young women aged 13 to 17 years who paid for it. In these circumstances, healthcare professionals felt hindered in recommending the vaccination to disadvantaged families: “*It is difficult to bring up the question of vaccination to youth if you can see they do not have the money to pay for it*, *or the family can*’*t afford to buy the vaccination for them as it is so expensive*, *it feels a little unethical to take this up with young people*” [Midwife, Sweden] [42]. Offering the HPV vaccine free at the point of delivery through a national school-based programme was considered advantageous in terms ability to reach the wider population: “*If one thinks about the whole community*, *then it is*, *of course*, *good*, *I think. So that everyone has the opportunity*… *Even those who don*’*t have the means*, *like those who have parents who wouldn*’*t pay*” [School nurse, Sweden] [43].

This important barrier to uptake was not evident in studies where the vaccine was offered free at the point of delivery.

#### Sexual mores

Social norms and values, particularly those relating to sexual debut and behaviour, shaped the views and actions of healthcare professionals, parents and young women in relation to the HPV vaccine.

This influenced the decisions of healthcare professionals about whether to recommend the HPV vaccine in healthcare settings [29-31,44,45]. Some young women were thought to be in greater need of protection: “*Well*, *you know*, *we try not to be judgmental*, *but we know there are certain* [*ethnic minority*] *populations where sexual activity with multiple partners is more common*, *more prevalent*…*we treat them frequently for various STDs*” [Pediatrician, USA] [30]. In other cases, healthcare professionals appeared reluctant to discuss sexually related information: “*There are just lots of docs who don*’*t like to talk about sex with their patients. That will be a huge barrier*” [Pediatrician, USA] [44]. This was especially pertinent for families with strong faith beliefs who were “*culturally more modest in terms of how they approach sexual issues*…*you*’*d see that in conservative Christian cultures as well*” [Pediatrician, USA] [30].

In this context, healthcare professionals noted that parental perceptions of adolescent sexual behaviour influenced parental decision-making: “*They don*’*t want their child to start talking about sex*… *or their parents don*’*t really know how to relay that information to them*” [Healthcare provider, USA] [32]. In one study, it was suggested parents would be receptive to the HPV vaccine: “*If we brought up the fact that there is a way of preventing even one out of four or five STDs*, *our parents are going to be banging on the door to get it at 11*, *12*, *13. I*’*m not worried about the discussion with our group*” [Clinician, USA] [45]. More commonly, resistance was anticipated due to connections with sexual activity [29,31,32,44,45]: “*HPV has so many other implications for parents*… *it*’*s one they fight you on*…*because you*’*re suggesting that their child is or will be sexually active soon*, *and they don*’*t want to hear that*” [Clinician, USA] [45]. In three studies, healthcare professionals reported that parents delayed vaccination of their daughter on this basis [31,32,44].

In contrast to delivery in healthcare settings, there was no evidence that perceptions of need by healthcare professionals affected their decisions to offer the HPV vaccine in school-based HPV vaccination programmes. However, there were concerns about the potential to adversely affect young women’s sexual health if young women assumed greater protection than is the case [42,43]: “*I haven*’*t heard it myself*, *but someone else heard from a girl that had been vaccinated that she was vaccinated against all sexually transmitted disease*” [Midwife, Sweden] [42]. In addition, some parental resistance to the HPV vaccine due to connections with sexual behaviour were reported [46,47]: “*We had quite a few phone calls*, *predominantly from* [*Asian*] *mothers*, *they were very concerned that by them okaying the needle*, *that was giving the daughters the green light to go and become sexually active*” [Teacher, Australia] [47].

Irrespective of delivery setting, the need for cultural sensitivity was highlighted: “*Cultures in which a girl must be a virgin at marriage*, *and so on. Clearly*, *it becomes very difficult to talk about multiple partners then. And one cannot*… *it*’*s like offensive*, *I think*” [School nurse, Sweden] [43]. This appeared to be an especially pertinent issue for parents with strong faith beliefs for whom sexual contact outside of marriage and multiple sexual partners were perceived to occur infrequently [47-49]: “*Coming from a Muslim background*… *We don*’*t have sex before marriage for example*, *so your first experience of these things are when you*’*re married and you stay in a relationship*… *because of that reason I*’*d probably say no*, *I wouldn*’*t bother with it with my two girls*” [Asian Muslim mother, UK] [49]. However, other parents recognised changing cultural norms and values, suggesting that their belief systems may differ to that of their daughter [47,48]: “*I went to the same school and I had a religious upbringing but a lot of my close friends who I grew up with are actually not religious now and they are living a different lifestyle than I am*… *my daughter may not grow up to live the way I do*” [Jewish mother, UK] [48].

The recommended age for vaccination was a concern for parents. Those who acknowledged that young women could be sexually active during adolescence appeared more accepting of the HPV vaccine [39,50]: “*I*’*d say probably* [*vaccination should happen at age*] *11 or 12 definitely before they have sex. I think while they*’*re still in early middle school. Once they get to the eighth grade and high school they*’*re already into puberty and thinking. The temptation is out there*” [Parent, USA] [39]. Others valued the protection afforded by HPV vaccination for situations that could not be predicted, such as the unknown sexual history of their partner [51-53]: “*Even though we try to practice that she*’*s only going to have sex with her husband*, *I*’*m a little more realistic than that. Even if she only does have sex with one man in her life*, *there*’*s no guarantee that he hasn*’*t had other partners and that he might not be a carrier*” [Mother, USA] [53]. In line with healthcare professionals’ accounts, some parents reported that they would delay vaccination to an age more closely aligned with their perception of sexual debut [45,48,54]: “*I*’*ve taught her not to have sexual relations at age 13*, *but at 16*, *it*’*s much more likely*” [Mother, USA] [54].

Parents also raised concerns that the HPV vaccine could invoke changes to sexual behaviour [38-40,51,54,55]: “*Because it would encourage my daughter to have sex and I wouldn*’*t want that*” [Mother, USA]. Parents raised concerns that the HPV vaccine might encourage earlier sexual debut, multiple sexual partners or complacency with regards to safe sexual health practices [49,56]: “*I think maybe something like that you might take for granted and not get screenings*, *because it still says to get screenings*, *but you might just think*, *obviously a 12 or 13 year old might be like oh*, ‘*I*’*ve had the vaccination now I*’*m fine*’ *and not get screenings*” [Mother, UK] [49]. Other parents dismissed these viewpoints [52,54]: “*I don*’*t think it*’*ll encourage my daughter to go out and have sex. I don*’*t want her to have sex now. She*’*s 14. I hope she has sex in the future and has kids and lives a normal life*, *but I don*’*t think it will encourage her to go act irrationally*” [Alaskan native father, USA] [52]. Personal experience of an HPV-related condition appeared to increase acceptance of the HPV vaccine [47-49,51,52,55,56]: “..*I thought what a fantastic thing* [*the vaccine*], *because I actually went to school with a girl who can*’*t have children because she*’*s got cervical cancer*, *and the reason she has cervical cancer is because she was very promiscuous when she was at school with me*” [Parent, Australia] [47].

There was evidence of parental discomfort in discussing sexually related information with their daughters [47,56,57]: “*So it*’*s easier to give it to younger children by saying* ‘*It*’*s to prevent cancer*’ *than saying to them* ‘*you*’*re having this because when you*’*re older you*’*re going to have sex and get all these horrible diseases*” [Mother, UK] [56]. Similarly, young women could also feel embarrassed [47,58]: “*We were just talking about it and she said* ‘*You don*’*t need it yet*, *do you*?’ *and I was like* ‘*of course not Mum*’ *and she was like* ‘*oh well*, *then we might just wait then*’ - *and I think I said* - …’ ‘*well does that mean I have to tell you when I need it*, *are you going to assume I*’*m sexually active*?’ *and she said* ‘*no*, *no*.’ *Well all I was saying is*, ‘*I want to have it now*, *because I don*’*t want to tell you when I need it*’ - *that is really awkward*” [Young woman, Australia] [47]. Others were anxious that vaccination might be negatively associated with their sexual behaviour [36-38]: “*If my family knows what this* [*HPV vaccine*] *is for and if I say I want it*, *they would think that I am fooling around*” [Young woman, Hong Kong] [36].

#### Trust

Issues of trust relate to the vaccine’s safety profile, the motives of pharmaceutical companies and whether it was recommended by a trusted source. The opinions of healthcare professionals were generally favourable towards the HPV vaccine to prevent cervical cancer [30,34,43-45,59-62]: “*Yes*, *definitely*… *It*’*s a very common virus*, *it causes quite a lot of disease and it can cause some very serious disease*, *in rare cases*, *but that can be prevented by the vaccine*” [General practitioner, UK] [59]. However, there was evidence for uncertainty in relation to the safety profile of the vaccine [31,43,44] and that early vaccination may mean that protection would not be maintained to the age of sexual debut [29,30,44].

Parental decision-making in favour of the vaccine was motivated by protection against HPV acquisition or the development of HPV-related conditions [33,50-52,54-56,63]. There was also evidence of implicit belief in vaccines overall, with views about childhood vaccination being transferred to the context of the HPV vaccine [33,48,49,55,56]: “*If there is a new vaccine to prevent a disease*, *we won*’*t oppose it because we are preventing a disease*” [Immigrant parent, USA] [39].

Despite generally positive perceptions to the HPV vaccine, parents expressed worries concerning side-effects and safety [38,39,48,49,51,53,56,63,64]: “*My first thought was*, *I am sendin*’ *my ten*-*year old to this clinic to put dead HPV cells in her. What if the HPV that they are shooting in her body*…*what if it comes to life*?” [African American, mother, USA] [65]. Such fears may be balanced against the benefits of protection from vaccination [48,49]: “*I just decided that it was more dangerous to get the diseases than to take the slight risk that there might have been side effects*” [Jewish mother, UK] [48].

Healthcare professionals suggested that parents with general ‘anti-vaccination’ beliefs were unlikely to make positive HPV vaccine decisions [30,40,46]: “*I have some* [*parents*] *that don*’*t want any vaccination*… *those I would not even broach the subject with*” [Paediatrician, USA] [30]. This was confirmed in studies reporting the views of parents, but only appeared to reflect the views of a minority [40,49,54]: “*Because she is not vaccinated*, *we use holistic medicine*” [Mother, USA] [54]. In one study, there was evidence that parental ‘anti-vaccination’ beliefs could influence their daughter: “*Well I don*'*t get immunizations. I*’*ve never had any. My dad believes in boosting our own immune system*, *not getting help*… *That*’*s what I see as the advantage of not getting* [*the vaccine*]” [Young woman, Australia] [47].

In the USA, parents appeared to be distrustful of pharmaceutical companies and governments providing the vaccine [52,53,64,66]: “*They* [*government*] *may not be telling the full story*, *which*, *of course*, *we found out about syphilis*, *we found out about AIDS*” [African-American participant] [66]. However, endorsement from a governmental source counteracted these concerns for other parents: “*At least* [*with*] *the* [*Centers for Disease Control and Prevention*] *you know they*’*ve got some validity behind their name that gives some validity to the drug maker because most drug makers I think are just out there to make a buck*” [Mother, USA] [67].

In countries with school-based HPV vaccination programmes, offering the vaccine through a trusted source appeared to reduce the burden of decision-making for parents: “*There may have been a covering information letter from the school*… *I suspect I read it*…*but I guess I must have somewhere made the decision when I saw that it was being offered to the general population that the girls would have it and I don*’*t think I did any further research at that point*” [Jewish mother, UK] [48]; “*If it hadn*’*t come to school it wouldn*’*t have crossed my mind to do it*… *It*’*s not* [*a decision you make*] *on an individual basis*, *and that* [*having it as school*] *makes you more comfortable*” [Parent, Australia] [47]. In one study, implicit trust in the healthcare system also appeared to influence young women’s acceptance of the vaccine: “*I think the people in charge*, *like Government*’*s health people have decided the jag* [*vaccine*] *is in our interest so I feel there*’*s no reason not to get it*” [Young woman, UK] [62].

However, insufficient or misleading information could also reduce trust and prevent young women receiving the HPV vaccine [47,68]: “*They pump all sorts of things into kids*… *Do they really know how it will affect them later*? *I don*’*t trust the government. Why would they need* [*the vaccination*] *when we didn*’*t get it*? *What aren*’*t they telling us*? *My sister showed me some articles about there being cancer in the vaccine*” [Parent, Australia] [47].

#### Consent

Because of the target age for HPV vaccination, parental consent is either essential or strongly preferred. A number of parents were worried that they could be excluded from the decision-making process [38,65]. Other parents appeared less engaged: “*I didn*’*t realise how ill*-*informed I am. You just sign off on all these forms*…” [Parent, Australia] [47].

Gaining consent in school-based HPV vaccination programmes, where the parent is unlikely to attend during the vaccination procedure, presented difficulties that were not raised in the healthcare-setting. Engaging some parents with the consent process could be challenging [46,47,69,70]: “*They couldn*’*t be bothered to read the form or fill it in*, *you know*, *motivate themselves enough to do it*, *but they always wanted the child to have it* [*the vaccine*] *as long as somebody bothered to knock on the door and say*, ‘*just sign on the dotted line*’. *It was just the process of the filling of the form in was just too much effort*” [School nurse, UK] [46]. Only one of 15 participating school nurses indicated willingness to vaccinate if a parental consent form had not been returned [61].

Differing levels of commitment to pursue consent were evident amongst the professionals involved [43,46,61]: “*It*’*s an offer. And it shouldn*’*t be our job to call and call again and again and invite them. I think that it*’*s actually the parent*’*s responsibility*, *together with the student* [*aged 12 to 13 years*]” [School nurse, Sweden] [43]. Targeting these parents with information, consent forms, and flexible HPV vaccination appointments was suggested [46,61,71]. This might be considered the responsibility of healthcare professionals or teaching staff at the school: “*You just need to circulate the information in different channels*, *like the school newsletter and*… *Daily notices to remind them to bring their* [*consent forms*] *back. I spoke at assembly*, *and they had year group meetings where the information was distributed and making sure the classes and teachers are informed of what*’*s happening*” [Teacher, Australia] [71].

In the UK, both primary care professionals and school nurses raised concerns about whether the school-setting was appropriate for assessing a young woman’s competence to receive the vaccine without parental consent [59,61,69]: “*If everybody is given 30 seconds to get in and get out*, *you can*’*t reasonably expect a nurse to make a decision in that time. Yes or no*? *They would have to make some sort of later appointment to speak to the young person and have a serious chat which in itself would then single them out from*, *you know if there was a mass queue*” [School nurse, UK] [69]. There was reluctance to challenge parents who actively refused consent for the HPV vaccine [46,61,69]: “*I would feel very uncomfortable about that because what we are trying to do is build up a relationship with parents here. I am asking parents to support the college in terms of rules*, *regulations*, *etc*., *and here I am then saying to parents*, ‘*Well in this case tough*, *what you say really does not count*.’ *It puts us in a very awkward position*” [Head teacher, UK] [69].

One school nurse justified her willingness to vaccinate in the face of parental refusal on the grounds of a clear health benefit: “*If I know this particular twelve year old girl is a risk of contracting an illness for which there is an injection that will prevent this illness then it is most definitely in her interest to give it to her*, *even if her parents don*’*t agree*” [School nurse, UK] [69]. Primary care professionals also acknowledged the difficulty of vaccinating without parental consent: “*Actually she*’*s being very responsible in coming*, *we want to encourage that. I still would feel extremely uneasy with going ahead with the immunisation although you begin to feel then that you are going against the fact she is trying to do something very responsible*” [General practitioner, UK] [59].

Irrespective of setting, the extent to which parents involved their daughters in decision-making about the HPV vaccine varied. Some parents reported making the decision, whereas others gave their daughter the freedom to choose [48,49,52,53,64,65]: “*I didn*’*t discuss it with the girls*, *I merely told them of my decision afterwards. I didn*’*t feel that they were in a position to make a decision for themselves*… *they are relatively sheltered and therefore it wouldn*’*t have been relevant to ask them what they thought*” [Jewish mother, UK] [48]; “*I would let the decision be up to her*, *but she should be informed. The more informed she is*, *the better off she*’*ll be making the decision*, *and she won*’*t feel forced by you*, *or anybody else*…” [White, female, USA] [65]. The relatively young age for vaccination may encourage some parents to make the decision on behalf of their daughter: “*I figured now is the best time because it*’*s a time that I can make the decision for her and I wanted to make sure she was protected before there was any chance of her becoming sexually active*” [Mother, USA] [53].

Very few studies examined young women’s views of the decision-making process [45,47,62]. There was some limited evidence to suggest young women were willing for decision-making to be the responsibility of parents and older adults [47,62]: “*I mean*… *We didn*’*t take much notice of the forms*, *and we handed it to our parents and they make the choice*… *It*’*s like your parents are the boss of you*, *sort of. You don*’*t choose*, ‘*oh I*’*m going to get a cervical cancer vaccination*” [Young woman, Australia] [47]. Young women may be disengaged: “*It doesn*’*t matter*, *I didn*’*t really care about it either way*” [Young woman, age 13, USA] [45]. Others may prefer greater involvement and to be better informed about the HPV vaccine and their parents’ decision: “*I did some research* (*on the net*) *at the time my mum said no. So I went in to learn more about it*… *She seemed to be thinking at the moment it is relatively new*, *and she didn*’*t have much confidence in that I needed it yet*” [Young woman, Australia] [47]. There was also limited evidence of young women exercising agency to prevent vaccination: “*Well she* [*my daughter*] *is just needle phobic*… *Even though I signed the consent forms*; *she just doesn*’*t turn up to them. Getting to this age makes it hard*, *too*, *because she just says* ‘*well it*’*s my body*, *my choice*, *and I*’*m not going*’ ” [Parent, Australia] [47].

## Discussion

### Key findings

**Figure 2:**
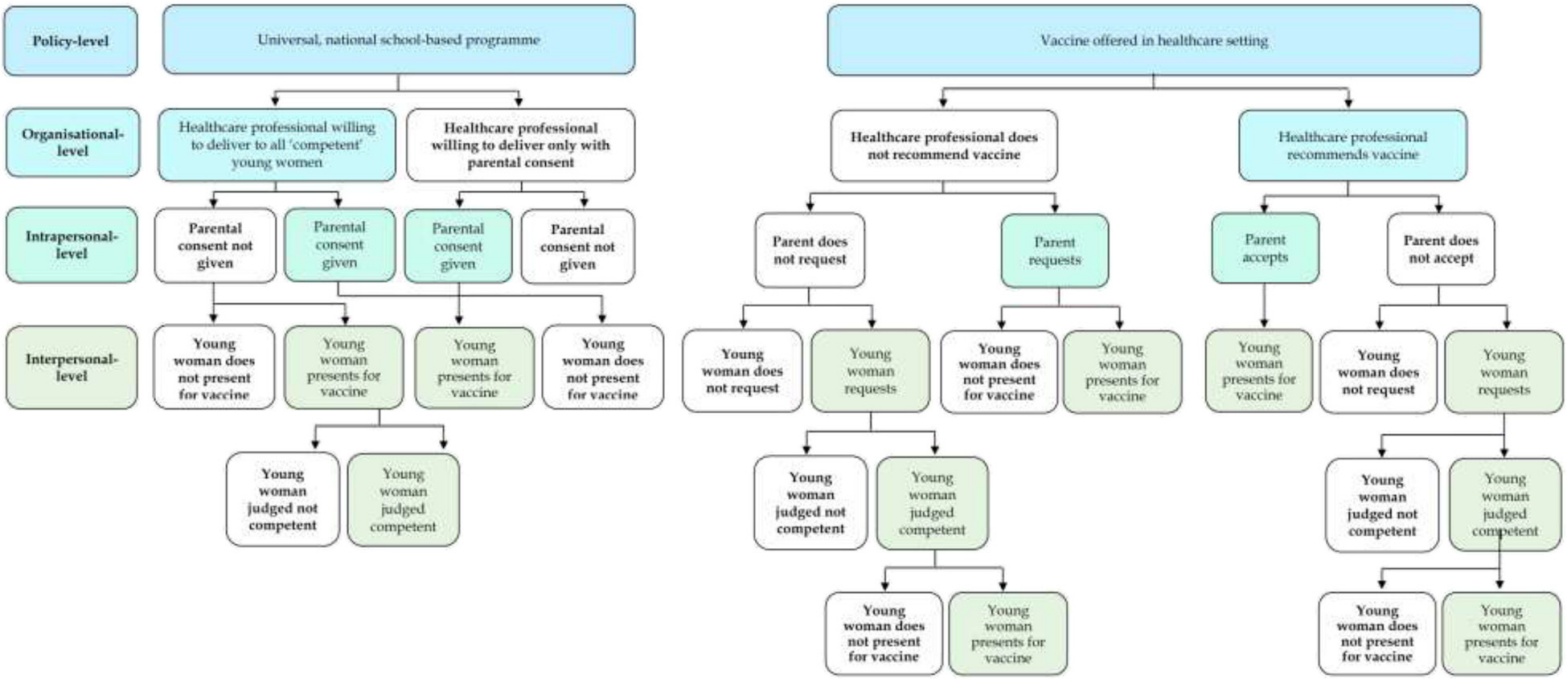
**Pathways of decision**-**making in relation to HPV vaccination of young women in high**-**income countries.**

The studies included in this review illustrate how a young woman’s access to the HPV vaccine is shaped by decisions at different levels of the socio-ecological model. This includes: the policy context; social norms and values, particularly in relation to sexual activity; the views and actions of healthcare professionals, and; parental consent. There is far less qualitative evidence of the role of young women in this important decision affecting their future sexual, physical and reproductive health. The stages at which decisions are made before young women are able to exercise any agency over whether they receive the vaccine are illustrated in Figure 2. Healthcare professionals’ decisions to recommend HPV vaccination, and the requirement for written parental consent, appear to be the most influential stages at which improvements to uptake of HPV vaccination programmes could be addressed.

### Policy decisions

Some of the most important decisions affecting access to the HPV vaccine are made at policy level, and relate to whether the vaccine is incorporated into the healthcare system, the financial arrangements for accessing the vaccine, and the arrangements for its delivery to the target population. A systematic review examining predominantly quantitative data from 22 studies (21 of which were conducted in the USA), reported that when cost is a factor in vaccine access it dominates as a barrier [72]. Similarly, the data examined for this qualitative systematic review and evidence synthesis highlight the importance of financial considerations to healthcare professionals and families where the costs of the vaccine are not met through a universal healthcare system. Studies investigating factors which affected decision-making at the policy level were underrepresented in the literature. Given the importance and cost of implementing initiatives such as HPV vaccination programmes, further research to understand factors that influence policy-level decision making should be considered.

### Healthcare recommendation

In healthcare settings, an important prompt to uptake appeared to be the decision for the healthcare professional to recommend vaccination. This has been reported in a systematic review, comprising predominantly quantitative primary studies, of barriers to HPV vaccination [73] and a statistical analysis linking HPV vaccine uptake with survey data from the US-Teen Survey [74]. The qualitative data also suggest the decision to recommend was influenced by concerns about safety of the HPV vaccine. However, value judgements about a young women’s likely sexual activity were also influential. Universal recommendation of the HPV vaccine needs to be incorporated into routine practice to ensure access for all eligible young women. Future studies could test whether patient reminder systems and computer prompts that remind healthcare professionals to recommend HPV vaccine to vaccine eligible young women increases uptake [75].

### Parental consent

The barrier of requiring a healthcare professional’s recommendation to receive the HPV vaccine was largely overcome in school-based programmes where the policy is to offer HPV vaccine universally to eligible young women. However, in this setting the requirement for written parental consent presented the greatest barrier to access. Healthcare professionals delivering the HPV vaccination programme could adapt implementation procedures to reach apparently ‘disengaged’ parents. This could be tested in existing HPV vaccination programmes by introducing strategies to introducing home visiting or chasing up consent forms if not returned [61,76].

### Young women’s autonomy

Vaccine safety, misperceptions of need based on sexual activity, and low perceptions of risk of HPV acquisition have been highlighted as barriers to positive HPV vaccine decision-making by young women [72]. However, the findings from this study suggest that young women are predominantly passive recipients of the HPV vaccine. Parents generally appeared keen to retain their role in decision-making on behalf of their daughters, and healthcare professionals appeared to reinforce this role.

Other combinations of decision-making about HPV vaccine were also identified. Vaccination may be sought by young women when parental consent has been actively refused or when a parental consent form has not been returned. Young women may also exercise their rights to refuse vaccination when parental consent has been granted, although there was little evidence for this within the qualitative studies included in the review. To address gaps in our understanding of this important process, further qualitative research should examine young women’s perceptions of, and involvement in, decision-making about the HPV vaccine. Further, healthcare professionals may require clear guidance and support in understanding how and when a young woman might be given the vaccine without parental consent.

### Social norms and values

A secondary objective of this review was to gain understanding of the factors contributing to inequalities in uptake of the HPV vaccination programme by ethnicity previously identified [4,7]. Corresponding with the findings of other studies [72,73], the importance of social norms and values, particularly sexual mores, were evident in the views of healthcare professionals, parents and young women. The qualitative evidence presented here suggests that some healthcare professionals avoided conversations with parents about the HPV vaccine if they perceived this to be culturally inappropriate. Lower likelihood of healthcare provider recommendation amongst ethnic minority parents has also been demonstrated from survey data of the United States National Immunisation Survey-Teen [74]. Young women are at risk of not receiving full levels of protection from the HPV vaccine if parents and healthcare professionals restrict access to the HPV vaccine, regardless of her needs and preferences. Development of strategies for healthcare professionals to overcome barriers to recommend HPV vaccination universally, regardless of cultural or religious group, are required.

Mistrust of the motivations of healthcare professionals in relation to the HPV vaccine was a pertinent issue for some minority ethnic groups. In the USA, the historical legacy of the mistreatment of African Americans in the Tuskegee syphilis experiment [77] (during which the USA Public Health Service studied the natural progression of untreated syphillis in rural African American men who thought they were receiving free health care) may contribute to levels of mistrust in contemporary populations. However, in other countries and in other populations there was also evidence of concerns about the safety of the vaccine by healthcare professionals as well as parents. Such concerns are exacerbated by negative media coverage. For example, in the UK the death of a young woman on the day she received the HPV vaccination received widespread media coverage and prompted anxiety in the wider population [78], despite her death being unrelated to the vaccine. Such stories reinforce anti-vaccine beliefs held by some sections of the population. Issues of trust require clear, accessible, and sometimes culturally appropriate, information about the HPV vaccination. Further, identifying community-engagement strategies to strengthen relationships between healthcare professionals and the populations they serve may be beneficial.

### Strengths and limitations

A systematic search of multiple databases was undertaken to identify all the relevant qualitative literature meeting the predetermined study criteria. Studies were not excluded on the basis of qualitative research method, or publication date, or population group. This has resulted in a comprehensive review capturing a range of perspectives resulting in a more complete picture in relation to decision-making for HPV vaccination of young women. The method of using qualitative synthesis within a socio-ecological framework enabled facilitators and barriers to be identified in relation to different stakeholders. This clearly illustrates the importance of targeting interventions at the appropriate level of decision-making, as well as identifying areas for further research.

Previously applied research methods to synthesis findings of qualitative studies were used to carry out the study [22,23]. The strengths of a qualitative synthesis include the possibility to reach conclusions based across common elements identified in heterogeneous studies. The results from a synthesis may be more accessible to a wider audience than if each of the primary studies had to be located individually. Further, a synthesis can provide a weight of evidence about particular issues. For example, few primary studies identified specifically addressed factors affecting uptake in minority ethnic populations. However, when the studies were combined more data on this topic was revealed.

There are some limitations. Studies not published in English were excluded and the findings reported therefore may be subject to English language publication bias. Although study selection was not limited by study design, few primary studies incorporating observational methodology were retrieved. Further, many of the studies reported views of stakeholders in relation to a hypothetical HPV vaccine. Therefore, primary studies have reported accounts provided by participants which may not reflect actual practice.

Thematic synthesis was undertaken by one reviewer (HF) with discussions held with the second reviewer (SA) as analysis progressed. The interpretation of the primary study findings, and consequently a different thematic framework, may have emerged if an additional reviewer had undertaken analysis at this stage. The subsequent exclusion of low- and middle-income countries from the study limits applicability of the findings within these settings. Further research to understand barriers and facilitators to HPV vaccination of young women in low- and middle-income countries is recommended. Finally, theoretical frameworks were underutilised in the primary studies. Of the 41 studies included in this review, only eight reported using theoretical models in the study [37,38,41,43,50,60,61,70].

## Conclusion

Our study findings show that access to the HPV vaccine is governed by parental influences, health professional recommendations, social norms and values, organisational factors and policy context. As such, interventions targeted only at young women are likely to be the least effective approach. Although young women are the main participants and beneficiaries of the HPV vaccination programme, their views are underrepresented in the literature. Future research efforts should develop context specific, culturally appropriate strategies that increase equitable access to the HPV vaccine.

## Abbreviations

ACIP: Advisory Committee on Immunisation Practices; HPV: Human Papillomvirus; UK: United Kingdom; USA: United States of America; WHO: World Health Organisation.

## Competing interests

The author’s declare that they have no competing interests.

## Authors’ contributions

SA and HF conceived and designed the study. All authors (HF, MH, CT and SA) are responsible for the reported research. HF and SA reviewed titles and abstracts and undertook data extraction. HF analysed the data and prepared the initial manuscript. All authors have made substantial contributions to interpreting the data, revising the manuscript and have given approval of the final version to be submitted.

## Pre-publication history

The pre-publication history for this paper can be accessed here:

http://www.biomedcentral.com/1471-2458/14/700/prepub

## Supplementary Material

Additional file 1 Search strategy applied to database. Click here for file
